# Clinical Evaluation of an Antigen Home Test Using Surface-Enhanced Raman Spectroscopy and Stacking Pad for SARS-CoV-2 Screening with Nasal and Salivary Swab Samples

**DOI:** 10.3390/diagnostics13050880

**Published:** 2023-02-24

**Authors:** Hyejin Ryu, Eunha Oh, Kyungjae Cha, Kina Kim, Soohyun Kim, Dohsik Minn

**Affiliations:** 1 Department of Diagnostic Immunology, Seegene Medical Foundation, Seoul 04805, Republic of Korea; 2 Immune Research Institute, Seegene Medical Foundation, Seoul 04805, Republic of Korea; 3 SG Medical, Inc., Seoul 05548, Republic of Korea

**Keywords:** SARS-CoV-2, antigen test, rapid test, COVID-19, diagnostics, self-testing, surface-enhanced Raman spectroscopy

## Abstract

This prospective study aimed to evaluate the performance of the InstaView COVID-19 (coronavirus diseases 2019) Antigen Home Test (InstaView AHT) which detects severe acute respiratory syndrome coronavirus 2 (SARS-CoV-2) antigens. In this test kit, surface-enhanced Raman spectroscopy was used, a stacking pad was inserted, and nasal swab and salivary swab samples were used simultaneously to improve performance. The clinical performance of the InstaView AHT was compared to that of RT-PCR using nasopharyngeal samples. The participants without any prior training were recruited and performed the sample collection, testing, and interpretation of the results by themselves. Of the 91 PCR-positive patients, 85 had positive InstaView AHT results. The sensitivity and specificity of the InstaView AHT were 93.4% (95% confidence interval [CI]: 86.2–97.5) and 99.4% (95% CI: 98.2–99.9). The sensitivity of the InstaView AHT was above 90% for all samples obtained from patients with Ct ≤ 20, 20 < Ct ≤ 25, and 25 < Ct ≤ 30 (100%, 95.1%, and 92.0%, respectively). The InstaView AHT can be used as an alternative to RT-PCR testing because of its relatively high sensitivity and specificity, especially when SARS-CoV-2 prevalence is high, and the availability of RT-PCR testing is limited.

## 1. Introduction

Severe acute respiratory syndrome coronavirus 2 (SARS-CoV-2) was first discovered in 2019 [1], and its spread has continued despite ongoing efforts to prevent transmission [2]. The standard method for diagnosing coronavirus disease 2019 (COVID-19) is to detect the presence of viral RNA by reverse transcription polymerase chain reaction (RT-PCR) using nasopharyngeal swabs (NPS) [3]. However, there are disadvantages to this testing method, such as restrictions on sample collection, technical complexity, long testing time, and high cost [4,5,6].

In contrast to the RT-PCR method, the rapid antigen test (RAT) is widely used because it does not require complicated techniques or large equipment and is convenient, rapid, and inexpensive [7,8,9]. In particular, the rapid antigen home test (RAHT) can be performed anywhere a sample can be collected for testing and reading; moreover, it does not require visits to hospitals or clinics and is convenient to use. However, RAT for SARS-CoV-2 diagnosis is generally known to be less sensitive than RT-PCR [10]. In addition, RAHT has the potential for inaccurate performance during self-sampling [9].

NPS, nasal swabs, oropharyngeal swabs, saliva, and sputum samples can be used to test for SARS-CoV-2 [11]. NPS samples can cause pain and nasal congestion owing to the invasive specimen collection method, which causes difficulties in specimen collection, particularly in patients with coagulopathy or children [12]. In comparison, nasal swab, saliva, and sputum samples are less invasive and convenient to collect because the expertise of professional medical staff is not required [13]. In addition, in a study comparing test results, NPS, nasal swabs from the front of the nasal cavity, and saliva samples showed similar sensitivities [14,15,16]. It has also been reported that sensitivity and specificity were high in samples collected from the nasal cavity and saliva at the same time, rather than in samples collected from the nasal cavity alone at the onset of the disease [17].

The InstaView COVID-19 Antigen Home Test (InstaView AHT; SG Medical, Seoul, Republic of Korea) used in this study was designed to increase sensitivity and specificity. First, nasal and salivary swab samples were used simultaneously during the sampling step. Then, gold nanoparticle complexes, using surface-enhanced Raman spectroscopy (SERS) technology, were used for the conjugate pad [18] and a stacking pad section [19] was inserted, which was different from existing products.

This prospective study aimed to compare the results of the InstaView AHT, which detects SARS-CoV-2 antigens using nasal and salivary swab samples self-collected, with the RT-PCR results of NPS samples collected by experts at the same time.

## 2. Materials and Methods

### 2.1. Patients and Specimens

Participants without any prior training were prospectively recruited to proceed on their own, from sample collection to testing, and interpretation of their results under minimal supervision by medical professionals. As for the positive patients, 99 volunteers were recruited from among those admitted to the Taereung Residential Treatment Center in Seoul. In the PCR retest before admission, five were negative and excluded; thus, 94 individuals were tested. The negative control group was comprised of 485 individuals who visited the Seegene Medical Foundation for a pre-departure examination. A case report was prepared for all samples and included their sex, age, date of symptom onset, date of sample collection, date of confirmation, and control reagent results.

### 2.2. Antigen Tests

InstaView AHT is an in vitro diagnostic medical device that tests for the presence of the coronavirus nucleocapsid protein (NP) antigen in nasal and salivary swab samples by immunochromatographic assay (ICA). 40 nm gold nanospheres were prepared according to the seed-growth nanoparticle synthesis method developed by Neus [20]. The synthesized gold nanoparticles were characterized by UV-visible spectroscopy and transmission electron microscopy (TEM), and the maximum absorption wavelength was 527 nm.

**Figure 1:**
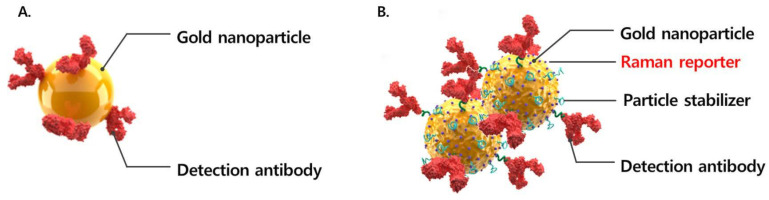
(**A**). Schematic representation of gold nanoparticle and detection antibodies. (**B**). Gold nanoparticle complex using surface-enhanced Raman spectroscopy. These particles form clusters, allowing more sensitive detection even in the presence of fewer antigens.

**Figure 2:**
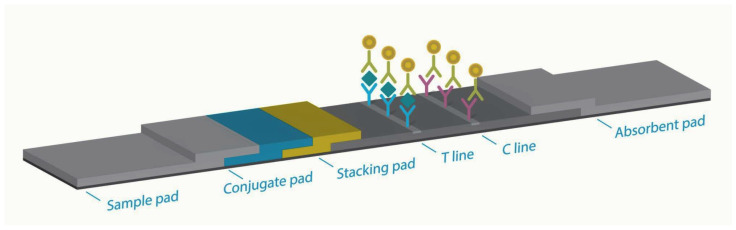
The InstaView COVID-19 Antigen Home Test device is attached in order of sample pad, conjugate pad, stacking pad, nitrocellulose membrane, and absorbent pad. The stacking pad exists between the conjugate pad and the membrane, and the sensitivity can be improved by increasing the reaction time between anti-SARS-CoV-2 antibodies conjugated gold nanoparticles present in the conjugate pad and the SARS-CoV-2 antigens.

It consists of a nitrocellulose membrane coated with a control line (C) and test line (T), a conjugate pad that can bind to the SARS-CoV-2 antigen in the sample, and a stacking pad. The control line is coated with goat anti-mouse antibodies, and the test line is coated with antibodies specific to the SARS-CoV-2 antigen. The conjugate pad contains gold nanoparticles coated with antibodies specific to the SARS-CoV-2 antigen. The sample is placed in the extraction solution, sufficiently mixed, and dropped into the sample inlet of the test device. If the sample contains the SARS-CoV-2 antigens, the antigens react with the gold nanoparticles to form nanoparticle complexes by SERS (Figure 1). It is designed to extend the antigen-antibody reaction time by adding a stacking pad between the conjugation pad and membrane (Figure 2). The antigen-antibody complexes react with the antibodies coated on the test line to form sandwich immune complexes, resulting in a red line.

All participants conducted the test according to the instructions after fully familiarizing themselves with the user manual and quick guide provided by the InstaView AHT product and the quick guide video provided in QR format. Participants first collected a nasal swab specimen. A sterile cotton swab was inserted up to about 1.5 cm into one nostril and turned along the wall of the nostril more than 5 times, and samples were also collected from the opposite nose with the same sterile swab. Saliva samples were collected by placing another sterile swab under the tongue in the mouth and rolling it at least 5 times to allow sufficient saliva to be absorbed into the sterile swab.

**Figure 3:**
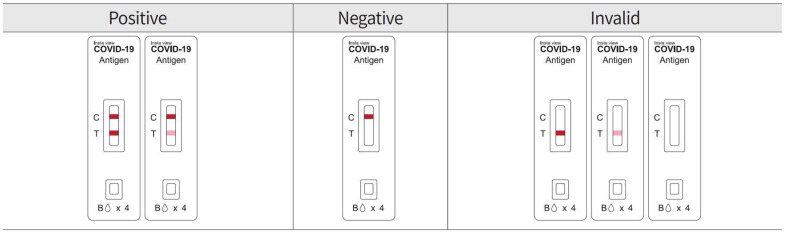
Interpretation of the test results of the InstaView COVID-19 Antigen Home Test.

The swabs collected from the nasal cavity and saliva were placed in the sample extraction solution for elution, and the results were visually confirmed 15 min after instillation into the sample inlet of the test device. If both the control line (C) and test line (T) appeared, the COVID-19 virus antigens were found in the sample, and the sample was judged to be positive for possible infection with COVID-19. If only the control line (C) appeared, no COVID-19 virus antigen was found in the sample, and it was judged as negative. If control line (C) did not appear, it was judged to be an invalid result (Figure 3). The LOD of InstaView AHT provided by the manufacturer was 5.938 × 10^4^ TCID_50_/_mL_, and the Ct value of the RdRP gene was 27.16.

### 2.3. Standard Reference RT-PCR

All participants underwent RT-PCR testing to detect the presence of the SARS-CoV-2 RNA-dependent RNA polymerase gene (RdRp) at the same time as the InstaView AHT. All specimens were collected using nasopharyngeal swabs, transported to the laboratory in a virus transport medium (VTM), and stored at 4 °C before and after testing, according to the guidelines reported by Hong et al. [21]. The Allplex™ 2019-nCoV Assay (Seegene, Seoul, Republic of Korea) was used according to the manufacturer’s instructions and the expert response from the Korea Centers for Disease Control and Prevention (COVID-19 Diagnosis Test Management Committee) [22]. The result was considered positive when the Ct (cycle threshold) value of genes was <33.5. The laboratory medicine specialist judged the results near the reference value with low viral titers.

### 2.4. Statistical Analysis

Sensitivity, specificity, positive predictive value (PPV), and negative predictive value (NPV) were evaluated based on the positive and negative results of the InstaView AHT and RT-PCR test methods and the statistical analysis was used by the chi-squared test. The differences in InstaView AHT sensitivity according to the period of the symptom onset date were evaluated using Fisher’s exact test. MedCalc^®^ Statistical Software version 20.2 (MedCalc Software Ltd., Ostend, Belgium) was used for all statistical analyses. Statistical significance was set at *p* < 0.001.

## 3. Results

### 3.1. Participants

From February to April 2022, 576 people were enrolled in the study at the Taereung Residential Treatment Center and Seegene Medical Foundation Departure Test Center. The RT-PCR results were 91 positive and 485 negative. The average age was 36.9 years (standard deviation 12.5), and 52.6% of the patients were women. The mean symptom onset in all RT-PCR-positive patients was 3.0 days, and the standard deviation was 1.5 days.

### 3.2. Comparison between InstaView AHT and RT-PCR

Of the 91 RT-PCR-positive patients, 85 had positive InstaView AHT results, and 482 of the 485 RT-PCR negative controls had negative InstaView AHT results (Table 1). The measured sensitivity, specificity, PPV, and NPV of InstaView AHT were 93.4% (95% confidence interval [CI]: 86.2–97.5), 99.4% (95% CI: 98.2–99.9), 96.6% (95% CI: 90.2–98.8), and 98.8% (95% CI: 97.4–99.3), respectively. The true negative and false positive rates of the InstaView AHT test measured in PCR-negative participants were 99.4% and 0.6%, respectively.

### 3.3. Comparison of Ct Values and Days of Symptoms of InstaView AHT Results

The sensitivity of the InstaView AHT was evaluated by dividing the Ct values of RT-PCR positive results into four groups: ≤20, 20 < Ct ≤ 25, 25 < Ct ≤ 30, and 30 < Ct. The sensitivity of InstaView AHT was >90% for all samples obtained from patients with Ct ≤ 20, 20 < Ct ≤ 25, and 25 < Ct ≤ 30, however, for patients with a Ct > 30, the sensitivity of InstaView AHT decreased to 75.0% (6/8) (Table 2).

The days of symptom onset in all RT-PCR-positive patients were within 5 days. There was no difference in the sensitivity of the InstaView AHT test according to the period from symptom onset to diagnosis (93.6% on days 1–2 and 93.2% on days 3–5, respectively). This suggests that 5 days of symptom onset is the optimal time to perform antigen testing (Table 2).

## 4. Discussion

In this study, 91 RT-PCR-positive and 485 RT-PCR-negative participants were prospectively collected from nasal and salivary samples to interpret the results. We evaluated the clinical performance of the InstaView AHT with the RT-PCR tests of NPS samples collected by experts. The clinical sensitivity and specificity of the InstaView AHT based on the RT-PCR test results were 93.4% (95% CI: 86.2–97.5) and 99.4% (95% CI: 98.2–99.9). These results met both the WHO criteria [3], which required a sensitivity of >80% and a specificity of 97–100%, and the Korean Ministry of Food and Drug Safety (MFDS) approval review criteria [23], which recommended a clinical sensitivity of ≥80% (with a lower limit of confidence interval of ≥70%), and a clinical specificity of ≥95% (with a lower limit of confidence interval of ≥90%).

Comparing the performance of RAHT and RT-PCR in previously reported studies [9,24,25,26,27], the sensitivity of RAHT ranged from 49–96%, and the specificity ranged from 82–100%. There was no significant difference in the diagnostic performance between the collected samples, and our study showed similar results. When the results were further subdivided according to the Ct value, they were found to be highly reliable for high viral loads, however, they were less sensitive when the viral load was low (Ct > 30). Shin et al. [26] reported a sensitivity of 73.33% at Ct > 25, and Kim et al. [27] reported a sensitivity of 63.6% at Ct > 30. In other previous studies, the sensitivity reached 98.4% when the viral load was high (<20 Ct or ≥10^7^ RNA copies/mL). However, as the viral load decreased, it was reported that the sensitivity decreased steadily to 36.7% at 10^4^ to <10^5^ RNA copies/mL and 7.5% at <10^4^ RNA copies/mL [28]. This can be considered a limitation of the RAT.

In this study, a sensitivity of 87.9% at Ct > 25 and 75.0% at Ct > 30 was achieved with better performance than that in previous studies using the following tools: first, the InstaView AHT used two samples, nasal swab, and salivary swab samples at the same time, and it is considered a test method that could increase the detection rate more than existing tests using a single sample. In a previous study comparing sensitivity between samples, Lindner et al. [29] reported positive and negative agreement rates of 90.6% and 99.2%, respectively, between antigen tests of self-collected nasal samples and NPS samples. Hanson et al. [14] reported positive and negative concordance rates of 93.8% and 97.8%, respectively, in tests using NPS and saliva samples, suggesting that using samples taken simultaneously from multiple anatomical sites could slightly increase the detection rate of SARS-CoV-2.

Second, in order to improve the low sensitivity, the InstaView AHT has the function of forming a nanoparticle complex when inserting a stacking pad. Gold nanoparticles are the most commonly used detection tool in lateral flow assays. The nanoparticles used in this device are gold nanoparticle complexes. The complex was formed by malachite green isothiocyanate (MGITC). These particles produce a colored readout that requires no development process for visualization and improves the sensitivity owing to the large number of nanoparticles per unit area (Figure 1) [18]. This kit was attached in the following order: sample pad, conjugate pad, stacking pad, nitrocellulose membrane, and absorbent pad. Unlike other products, a stacking pad is designed to improve sensitivity by increasing the reaction time between the SARS-CoV-2 antigens present on the conjugate pad and SARS-CoV-2 antibodies conjugated with gold nanoparticle complexes [19]. The stacking pad increases the reaction efficiency by increasing the reaction time between nanoparticles and samples. Clustered nanoparticles can increase sensitivity by giving the same effect as multiple nanoparticles reacting to one antigen. In addition, Raman signals are greatly amplified by “hot spots” generated between nanoparticles. Thus, it shows better performance than the other products by using two samples, SERS, and a stacking pad.

This study has some limitations. First, the average age of the participants was low, and it did not reflect the entire population distribution. Second, the detection targets of the InstaView AHT and RT-PCR are not the same as nucleocapsid and RdRp, respectively. Third, the samples of InstaView AHT and RT-PCR test were different. Fourth, as we could not find individuals 5 days after symptom onset, we could not obtain sensitivity in patients with long-term symptoms, as this was a prospective study that included patients who were hospitalized immediately after RT-PCR positivity. However, while most previous studies were retrospective studies using archived samples, this study was a prospective study, therefore, the data reflected are believed to be more realistic.

## 5. Conclusions

In conclusion, the InstaView AHT showed excellent performance as a kit for detecting SARS-CoV-2 antigens. Therefore, when RT-PCR testing is limited, COVID-19 results can be easily and quickly confirmed through self-collection.

## Figures and Tables

**Table 1:** Comparison of InstaView COVID-19 Antigen Home Test and RT-PCR results.

		RT-PCR		Total	*p*-Value *
		Positive	Negative		
InstaView COVID-19 Home Test	Positive	85	3	88	<0.0001
	Negative	6	482	488	
Total		91	485	576	

* Chi-squared test.

**Table 2:** Clinical performance analysis of InstaView COVID-19 Antigen Home Test according to Ct values and days of symptom onset.

Ct Values	InstaView COVID-19 Antigen Home Test		Sensitivity (%) (95% CI)
	Positive	Negative	
Overall	85	6	93.4 (86.2–97.5)
RT-PCR Ct values			
Ct ≤ 20	17	0	100 (80.5–100)
20 < Ct ≤ 25	39	2	95.1 (83.5–99.4)
25 < Ct ≤ 30	23	2	92.0 (74.0–99.0)
30 < Ct	6	2	75.0 (34.9–96.8)
Days after symptom onset			
1–2	44	3	93.6 (82.5–98.7)
3–5	41	3	93.2 (81.3–98.6)

Abbreviations: Ct, cycle threshold; CI, confidence interval.

## Data Availability

The data that support the findings of this study are available from the corresponding author upon reasonable request.
